# A Proposal for a Simple Subclassification of Advanced Hepatocellular Carcinoma in Systemic Treatment

**DOI:** 10.3390/cancers16223797

**Published:** 2024-11-12

**Authors:** Norihiro Imai, Takafumi Yamamoto, Kazuyuki Mizuno, Shinya Yokoyama, Kenta Yamamoto, Takanori Ito, Yoji Ishizu, Teiji Kuzuya, Takashi Honda, Tetsuya Ishikawa, Hiroki Kawashima

**Affiliations:** 1 Department of Gastroenterology and Hepatology, Nagoya University Graduate School of Medicine, 65 Tsurumai-cho, Showa, Nagoya 466-8550, Japan; 2 Department of Gastroenterology and Hepatology, Fujita Health University, 1-98 Kutsukake-cho, Toyoake 470-1192, Japan

**Keywords:** advanced hepatocellular carcinoma, immune checkpoint inhibitor, vascular invasion, extrahepatic metastasis

## Abstract

Objectives: This study focused on the presence or absence of vascular invasion and extrahepatic metastasis in hepatocellular carcinoma (HCC) and examined their impact on systemic treatment outcomes. Methods: We retrospectively analyzed 362 patients with unresectable HCC who received first-line systemic therapy. The prognostic evaluation was based on the presence of vascular invasion and extrahepatic metastasis at the time of treatment initiation. Results: Patients with vascular invasion or extrahepatic metastasis (advanced group) had significantly worse outcomes than those without these features (intermediate group), with median survival times of 434 and 658 days, respectively. Further subdivision of the advanced group into three categories—patients with only extrahepatic metastasis (m group, n = 77), patients with only vascular invasion (v group, n = 78), and patients with both vascular invasion and extrahepatic metastasis (vm group, n = 52)—revealed that the m group had significantly better outcomes than those in the other two groups, with median survival times of 649, 323, and 187 days, respectively. A comparison of the clinical backgrounds among the three groups demonstrated that the m group had significantly better liver function at the time of treatment initiation than that in the other two groups. Multivariable analysis, including performance status, Child–Pugh score, and the use of immune checkpoint inhibitors as first-line therapy, identified the m group as an independent and significant prognostic factor (hazard ratio, 0.50). Conclusions: Unresectable HCC with extrahepatic metastasis and no vascular invasion represents a novel staging category for systemic treatment.

## Simple Summary

This study aimed to understand how specific characteristics of liver cancer, specifically the spread of cancer to blood vessels or other parts of the body, affect treatment outcomes. By analyzing data from 362 patients who received first-line treatment for liver cancer that could not be surgically resected, the researchers identified patterns that could assist in predicting how well patients will respond to treatment. Patients whose cancer had spread only to other parts of the body lived longer than those whose cancer had spread to the blood vessels or both areas. These findings suggest that patients whose cancer had spread only outside the liver may represent a distinct group that could benefit from specific treatment strategies. This new insight may help doctors tailor treatments for liver cancer and improve patient outcomes.

## 1. Introduction

Primary liver cancer ranks as the sixth most commonly diagnosed cancer globally and is the third leading cause of cancer-related mortality. The primary types include hepatocellular carcinoma (HCC), which constitutes 75–85% of cases, and intrahepatic cholangiocarcinoma, comprising about 10–15%. Other, rarer forms of liver cancer also exist [1]. HCC typically develops from hepatocytes, often in patients with chronic liver conditions like hepatitis B virus (HBV) or C virus (HCV) infections, alcohol abuse, or metabolic dysfunction-associated steatotic liver disease. The risk factors for HCC vary geographically, with chronic HBV infections and aflatoxin exposure prevalent in high-risk areas such as China, the Republic of Korea, and sub-Saharan Africa, while HCV infection is more prominent in regions like Japan, Italy, and Egypt. Additional risk factors include aflatoxin-contaminated foods, heavy alcohol use, obesity, type 2 diabetes, and smoking [1,2,3,4,5].

Symptoms of HCC include fatigue, abdominal pain or swelling, unexplained weight loss, and jaundice; however, most patients are asymptomatic in the early stages, underscoring the need for regular screening through ultrasonography, computed tomography (CT), magnetic resonance imaging (MRI), and blood tests in those with chronic liver disease [6,7]. Globally, the importance of nonviral risk factors is rising due to the decline in HBV and HCV prevalence, alongside a rise in obesity and diabetes. However, eliminating viral hepatitis remains crucial, as HBV and HCV infections contribute to 56% and 20% of liver cancer deaths worldwide, respectively [1]. Treatment for HCC is tailored based on cancer stage, liver function, and the patient’s overall health condition. Treatment options for early-stage HCC include ablation, resection, radiation, transarterial chemoembolization, and liver transplantation. However, the treatment options for advanced-stage HCC are generally limited to systemic therapy [8,9,10].

Advances in systemic treatment have resulted in significant changes in the treatment outcomes of unresectable HCC [11,12,13]. With the advent of immune checkpoint inhibitors and other tyrosine kinase inhibitors, multiple drug treatments have become available, and the indications for each are rapidly evolving [14,15,16,17,18,19,20,21,22]. The Barcelona Clinic Liver Cancer (BCLC) staging system is widely used as a treatment guideline for HCC [23]. In this classification, advanced HCC (BCLC-C) is defined as “patients presenting with vascular invasion or extrahepatic spread who are still relatively fit,” with an expected survival period of about 2 years. However, in clinical practice, the prognosis of advanced HCC varies significantly. At the same time, some patients live well beyond 2 years, while many have a prognosis of less than 6 months [24,25,26]. Therefore, a precise subclassification of advanced HCC based on treatment outcomes is highly desirable. In this study, we focused on the presence or absence of vascular invasion and extrahepatic spread when initiating systemic treatment. We identified a simple subclassification associated with treatment outcomes.

## 2. Materials and Methods

### 2.1. The Study Population

We retrospectively collected clinical data from patients who received first-line systemic treatment between 2011 and 2024 at the Nagoya University Hospital in Aichi, Japan. Baseline characteristics, including age, sex, underlying liver disease, laboratory data, and tumor-specific factors, such as the duration of drug administration, tumor stage based on the BCLC classification, macrovascular and portal vein invasion, and extrahepatic spread, were retrospectively assessed. This study was conducted in accordance with the Declaration of Helsinki and approved by the Institutional Review Board of Nagoya University Hospital (No. 2021-0247).

### 2.2. HCC Diagnosis

HCC was primarily diagnosed using hemodynamic imaging techniques, including contrast-enhanced CT, gadolinium ethoxybenzyl diethylenetriamine pentaacetic acid (Gd-EOB-DTPA)-enhanced MRI, and/or contrast-enhanced ultrasonography with perflubutane [27,28,29,30]. Pathological diagnosis was performed only in inconclusive cases. Cases with imaging evidence of invasion into the portal vein, hepatic vein, or bile duct were classified as vascular invasion cases. Patients with tumor invasion in the portal or hepatic veins were further classified according to the level of the affected branch. The presence, absence, and classification of vascular invasion were determined based on the radiologist’s report. The presence of extrahepatic metastasis was primarily assessed using contrast-enhanced CT of the thorax and abdomen. In cases where intrahepatic lesions alone did not align with clinical findings, including tumor markers, additional imaging such as PET-CT, gallium scintigraphy, or head MRI was performed.

### 2.3. Liver Function Assessment

Liver function was assessed using the Child–Pugh classification and the albumin–bilirubin (ALBI) score. In brief, the ALBI score was calculated based on laboratory data using the following formula: ALBI score = log10 bilirubin (µmol/L) × 0.66 + albumin (g/L) × −0.085 [31].

### 2.4. Systemic Therapy

Patients continued systemic therapy until one of the following occurred: confirmed disease progression, unacceptable adverse events, withdrawal of consent, or a physician’s decision to discontinue treatment based on the patient’s condition and clinical data. When it became challenging to continue primary treatment or when progression was observed on imaging, patients were switched to a possible second-line or later treatment. Decisions to add local treatment during systemic treatment were primarily at the discretion of the treating physician. Sorafenib was administered until February 2018 [17,18], lenvatinib between March 2018 and October 2020 [19], atezolizumab plus bevacizumab from November 2020 [20,21], and tremelimumab plus durvalumab from January 2023 [22]. However, the choice of agent was also influenced by complications. The systemic therapy regimens included: 400 mg of sorafenib taken orally twice daily; 12 mg of lenvatinib daily for patients weighing 60 kg or more, or 8 mg daily for those weighing less than 60 kg; 1200 mg of atezolizumab combined with 15 mg/kg of bevacizumab administered intravenously every three weeks; and 300 mg of tremelimumab given intravenously once, followed by 1500 mg of durvalumab administered intravenously every four weeks. Patients who began systemic treatment were primarily monitored through outpatient visits at our hospital. For those transferred to other hospitals during the follow-up period, outcome information was obtained from the new hospital. Cases in which no information could be obtained from the receiving hospital, or where patients ceased attending follow-up appointments, were treated as censored data.

### 2.5. Assessment of Treatment Effects and Adverse Events

Treatment effects were generally evaluated every 2 to 4 months using contrast-enhanced CT or Gd-EOB-DTPA-enhanced MRI, in accordance with the RECIST and mRECIST criteria [32]. Additionally, tumor markers such as alpha-fetoprotein and des-γ-carboxy prothrombin were assessed at each patient visit. Adverse events were graded using the Common Terminology Criteria for Adverse Events version 5.0 [33]. When CTCAE grade 3 or higher adverse events occurred, drug therapy was suspended until adverse events improved to grade 2 or lower, at which point the decision to resume treatment was considered. Drugs were also discontinued or interrupted according to the manufacturer’s instructions.

### 2.6. Statistical Analysis

Categorical variables, described as numbers, were compared using the chi-square test, and continuous variables, described as means (minimum to maximum), were compared using a one-way analysis of variance. Multiple comparisons were performed using Tukey’s multiple comparison test. Overall survival (OS) was assessed using the Kaplan–Meier method, with comparisons made using the log-rank test. Cox proportional hazard models were employed to estimate hazard ratios and 95% confidence intervals (CIs). A P-value of less than 0.05 was considered statistically significant for all tests. Statistical analyses were conducted using GraphPad Prism 9 (GraphPad Software, San Diego, CA, USA), R (version 4.1.2, R Foundation for Statistical Computing, Vienna, Austria; http://www.R-project.org/, accessed on 1 April 2024), and EZR (Saitama Medical Center, Jichi Medical University, Saitama, Japan), which is a graphical user interface for R [34].

## 3. Results

**Figure 1:**
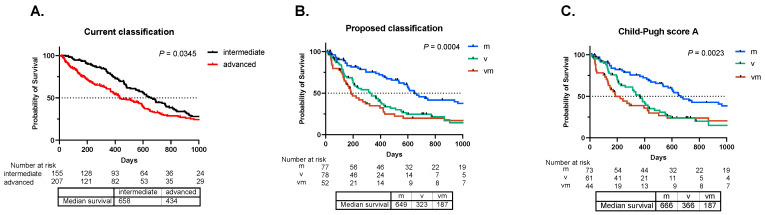
Comparison of the current and proposed classifications for advanced hepatocellular carcinoma undergoing systemic treatment. After first-line systemic treatment, overall survival (OS) was analyzed using the Kaplan–Meier method and compared using the log-rank test. (**A**) illustrates the comparison of OS between the 207 patients with vascular invasion or extrahepatic metastasis (advanced group) and 155 patients without these features (intermediate group). (**B**) presents an OS comparison based on the proposed classification as follows: 77 patients with extrahepatic metastasis only (m group), 78 patients with vascular invasion only (v group), and 52 patients with both vascular invasion and extrahepatic metastasis (vm group). (**C**) compares OS among patients with a Child–Pugh score of A across the three groups.

Approximately 362 patients with HCC who received first-line systemic therapy were enrolled during the study period. Table 1 presents a summary of the patient information, including demographic data, clinical characteristics, and relevant baseline parameters. The breakdown of first-line treatments was as follows: sorafenib, two hundred and seven cases; lenvatinib, seventy-two cases; atezolizumab plus bevacizumab, seventy-five cases; and durvalumab plus tremelimumab, eight cases. By applying the “current” BCLC classification, 207 patients with either vascular invasion or extrahepatic metastasis (advanced group) had significantly worse prognoses than the prognosis of the 155 patients without these features (intermediate group), with a median survival of 434 days vs. 658 days (Figure 1A).

Further classification of the advanced group into three subgroups—77 patients with only extrahepatic metastasis (m group), 78 patients with only vascular invasion (v group), and 52 patients with both vascular invasion and extrahepatic metastasis (vm group)—demonstrated that the m group had a significantly better prognosis than the other two groups, with a median survival time of 649 days in the m group, 323 days in the v group, and 187 days in the vm group (Figure 1B). The five-year survival rates according to the proposed subclassification were as follows: 20.7% for the m group, 9.6% for the v group, and 12.8% for the vm group. Even among patients with a Child–Pugh score of A for liver function at the initiation of systemic therapy, those in the m group had a significantly better prognosis than those in the other two groups (Figure 1C). We hypothesized that the significantly better OS observed in the m group might be attributed to radical therapeutic interventions aimed at controlling extrahepatic lesions. However, during the observation period, only four patients received radical local therapy for extrahepatic lesions—one patient underwent proton beam therapy, one received stereotactic radiotherapy, one was treated with ablation, and one with surgical resection. This indicates that the number of patients who underwent radical local treatment for extrahepatic lesions was limited. These results reveal distinct subgroups of advanced HCC, potentially indicating different prognoses in response to first-line systemic treatments.

A comparison of the clinical backgrounds of the three groups revealed that the m group had significantly better liver function at the time of treatment initiation than the function in the other two groups (Table 2). In addition, intrahepatic lesions were substantially larger in the v group than in the m group. Although no significant differences in progression-free survival among the three groups were observed, the time to treatment failure was significantly longer in the m group than in the other two groups, suggesting better treatment tolerance in the m group (Table 2).

**Figure 2:**
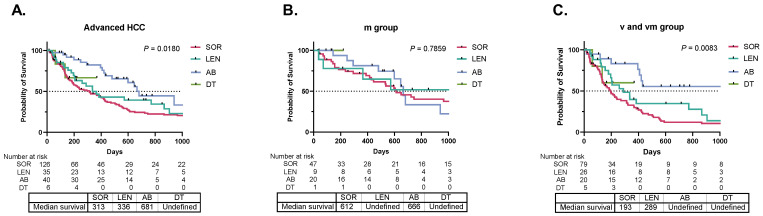
Comparison of initial systemic treatment regimens for advanced hepatocellular carcinoma. Overall survival (OS) following first-line systemic treatment among 207 patients with either vascular invasion or extrahepatic metastasis (advanced group) was analyzed using the Kaplan–Meier method and compared using the log-rank test. (**A**) displays the OS comparison between one hundred and twenty-six patients treated with sorafenib, thirty-five patients treated with lenvatinib, forty patients treated with atezolizumab plus bevacizumab, and six patients treated with durvalumab plus tremelimumab. (**B**) illustrates the OS comparison based on the initial systemic treatment among the 77 patients with only extrahepatic metastasis (m group). (**C**) illustrates the OS comparison based on the initial systemic treatment among 78 patients with only vascular invasion (v group) and 52 patients with both vascular invasion and extrahepatic metastasis (vm group).

In a study of 207 patients who received first-line systemic treatment for advanced HCC, those treated with atezolizumab plus bevacizumab had a significantly improved prognosis (Figure 2A). A significant difference was observed in OS between patients treated with sorafenib and those treated with atezolizumab plus bevacizumab, with a *p*-value of 0.0014. However, in the m group, no significant difference was noted in the prognosis based on the type of initial systemic treatment (Figure 2B). Notably, the atezolizumab plus bevacizumab treatment demonstrated significantly better outcomes in the v and vm groups than other systemic treatments (Figure 2C). The comparison between the treatment groups revealed no significant difference in OS between patients treated with lenvatinib and those treated with atezolizumab plus bevacizumab, with a *p*-value of 0.05. However, a significant difference was found between the sorafenib group and the atezolizumab plus bevacizumab group, with a *p*-value of 0.0006. These results suggest that the improved prognosis following atezolizumab plus bevacizumab treatment in advanced HCC is primarily attributable to the effects of the drugs on the vm and vm groups.

In the univariate analysis of factors associated with survival, the m group, performance status, Child–Pugh score, and initial treatment with immune checkpoint inhibitors were identified as significant (Table 3). In the multivariable analysis, which included these factors, the m group was identified as an independent and significant factor (hazard ratio 0.50, Table 3). Taken together, patients with advanced HCC with only extrahepatic metastases represent a distinct subgroup in terms of prognosis after first-line systemic treatment.

## 4. Discussion

We focused on the presence or absence of vascular invasion and extrahepatic metastasis at the time of first-line systemic therapy for HCC. We identified that the prognosis could be broadly categorized into three groups. Among these, patients with extrahepatic metastasis but no vascular invasion (group m) had a better prognosis after the introduction of systemic therapy than those in the other two groups. Applying this simple subclassification at the start of systemic therapy can help guide appropriate treatment decisions.

The prognosis of HCC is significantly influenced not only by the stage of the cancer but also by liver function, performance status, and comorbidities, making individual patient evaluation essential. The BCLC system is commonly used to stage HCC, and each stage provides a general prognostic prediction [23]. For cases classified as BCLC-C with vascular invasion or extrahepatic metastasis, the expected survival time is approximately 2 years and systemic therapy is recommended. Despite recent advancements in systemic therapy for HCC, leading to trends in prolonged OS, the prognosis for HCC cases classified as BCLC-C varies widely, with many cases having a survival time of less than 6 months [21,22,23,24].

In this study, immune checkpoint inhibitor administration was a significant factor for OS in univariate analysis, consistent with previous studies [20,21,22]. However, as the study included a period when no systemic therapies beyond sorafenib were approved, the results may also reflect the influence of subsequent treatments following primary therapy.

While there was no difference in PFS between the m group and other groups, a significant difference was observed in TTF. This suggests that intrahepatic lesion progression with vascular invasion, including diminished hepatic reserve, may serve as a bottleneck to continued treatment. Therefore, the favorable prognosis in the m group may have been due to the absence of vascular invasion, allowing for more options for sequential medical therapies or additional local treatments aimed at controlling intrahepatic lesions. However, given the retrospective nature of this study, it is challenging to assess the differences between patients with and without additional or sequential treatment after the initiation of the systemic therapy. Given the favorable prognosis observed in the m group, further studies are warranted to confirm the potential clinical benefits of adding locoregional treatments for intrahepatic lesions in this subgroup.

Recently, new subclassifications such as the Bolondi classification and Kinki criteria have been proposed and widely applied in clinical practice for HCC cases classified as BCLC-B (intermediate HCC) [35,36]. However, investigations into the subclassification of BCLC-C cases are limited. Based on our results, we propose a new subclassification for predicting the prognosis of advanced HCC when introducing systemic therapy, focusing on the presence or absence of vascular invasion and extrahepatic metastasis. Owing to the retrospective study design, we could not determine the underlying cause of the improved prognosis in the m group. However, we speculate that the presence of vascular invasion may aggravate liver function, leading to an insufficient relative dose intensity for systemic therapy and/or reduced capacity for subsequent treatments.

While portal vein invasion has been established as a clear poor prognostic factor in prior prospective studies of systemic pharmacotherapy, studies specifically focusing on the subclassification of extrahepatic metastasis without vascular invasion remain scarce [14,15,16,17,18,19]. Future prospective studies on systemic therapy would benefit from incorporating and randomizing patients according to this subclassification.

We believe that our new subclassification offers a more reliable basis for prognosis and patient counseling at the initiation of drug therapy for unresectable hepatocellular carcinoma. Our findings further underscore the importance of controlling intrahepatic lesions in the pharmacological management of this condition and provide a rationale for a pharmacotherapy-based multidisciplinary treatment approach.

This study has three main limitations: (1) As a single-center, retrospective observational study conducted in Japan with limited number of cases, international multicenter validation is desirable. (2) The extended study period introduces potential influences from changes in treatment protocols and patient demographics. (3) This study did not comprehensively examine post-initial pharmacotherapy treatments in detail.

As this was a single-center, retrospective observational study, further validation in a multicenter setting is necessary. We hope that this proposal contributes to the reclassification of advanced HCC.

## 5. Conclusions

Advanced HCC with extrahepatic metastases but no vascular invasion represents a new staging category for the initiation of systemic treatments.

## Figures and Tables

**Table 1:** Patient characteristics.

Characteristics	
Number of patients	362
Age (year)	70 (34–92)
Sex (M/F)	297/65
Etiology (B/C/NBNC)	66/115/181
PS (0/1/2)	275/80/7
Systemic treatment history	none
Medication (SOR/LEN/AB/DT)	207/72/75/8
AFP (U/mL)	24,914 (1–3,610,200)
DCP (mAU/mL)	16,995 (0–603,470)
T-bil (mg/dL)	0.97 (0.3–5.1)
Alb (g/dL)	3.6 (2.2–4.8)
CPS (A/B/C)	320/42/0
HCC size (mm)	33.8 (0–182)
BCLC stage (A/B/C)	19/109/234
Extrahepatic metastasis (%)	35.6 (129/362)
Lung metastasis (%)	20.4 (74/362)
Lymph node metastasis (%)	7.5 (27/362)
Bone metastasis (%)	6.1 (22/362)
Other metastasis (%)	9.4 (34/362)
Vascular invasion (%)	35.9 (130/362)
Portal vein invasion (Vp1/Vp2/Vp3/Vp4)	2/34/49/20
Hepatic vein invasion (Vv1/Vv2/Vv3)	1/19/17
Bile duct invasion (%)	5.2 (19/362)

PS, performance status; SOR, sorafenib; LEN, lenvatinib; AB, atezolizumab–bevacizumab; DT, durvalumab–tremelimumab; AFP, alpha-fetoprotein; DCP, des-gamma-carboxy prothrombin; T-bil, total-bilirubin; Alb, albumin; CPS, Child–Pugh score.

**Table 2:** Comparison of patient characteristics across the three groups.

	m	v	vm				
Number of patients	77	78	52	Fisher’s exact test *p* value			
Sex (M/F)	64/13	62/16	43/9	0.8740			
Etiology (B/C/NBNC)	17/21/39	16/22/40	11/16/25	0.9930			
PS (0/1/2)	59/17/1	56/19/3	33/18/1	0.4400			
Medication (SOR/LEN/AB/DT)	47/9/20/1	44/18/12/4	35/8/8/1	0.2190			
					Turkey’s test *p* value		
				one-way ANOVA *p* value	m vs. v	m vs. vm	v vs. vm
Age (year)	72 (34–91)	69 (40–92)	67 (37–87)	0.1654	-	-	-
PFS (day)	117 (13–2704)	95 (14–2129)	56 (7–966)	0.0503	-	-	-
TTF (day)	216 (4–4071)	119 (6–1146)	88 (7–2370)	0.0024	0.0067	0.0108	0.9850
AFP (U/mL)	22 (1–382,175)	289 (2–656,984)	1413 (2–3,610,200)	0.1088	-	-	-
DCP (mAU/mL)	160 (0–603,470)	2392 (0–500,000)	1431 (10–490,380)	0.1410	-	-	-
PT-INR (ratio)	1.05 (0.91–1.33)	1.08 (0.93–1.57)	1.08 (0.96–1.31)	0.1361	-	-	-
T-bil (mg/dL)	0.7 (0.4–1.6)	0.9 (0.3–5.1)	0.9 (0.4–4.3)	0.0485	0.0529	0.0079	0.6243
Alb (g/dL)	3.8 (2.6–4.8)	3.5 (2.2–4.4)	3.6 (2.4–4.5)	0.0040	0.0048	0.0449	0.8924
ALBI	−2.42 (−3.24–−1.51)	−2.20 (−2.93–−1.16)	−2.21 (−3.21–−1.15)	0.0004	0.0009	0.0060	0.9789
CPS	5 (5–7)	6 (5–8)	6 (5–9)	0.0030	0.0076	0.0140	0.9940
HCC size (mm)	23 (0–170)	33 (5–182)	23 (5–180)	0.0075	0.0050	0.3731	0.3006

m group, patients with only extrahepatic metastasis; v group, patients with only vascular invasion; vm group, patients with both vascular invasion and extrahepatic metastasis; PS, performance status; SOR, sorafenib; LEN, lenvatinib; AB, atezolizumab–bevacizumab; DT, durvalumab–tremelimumab PFS, progression-free survival; TTF, time to treatment failure; AFP, alpha-fetoprotein; DCP, des-gamma-carboxy prothrombin; PT-INR, prothrombin time-international normalized ratio; T-bil, total-bilirubin.

**Table 3:** Univariate and multivariable analysis of overall survival following first-line systemic treatment for hepatocellular carcinoma.

	Univariate Analysis			Multivariable Analysis		
Parameter	Hazard Ratio	95% CI	*p* Value	Hazard Ratio	95% CI	*p* Value
m group	0.50	0.35–0.71	0.0001	0.55	0.38–0.80	0.0021
Age	0.92	0.64–1.32	0.6711	0.84	0.56–1.25	0.4051
Sex	0.76	0.50–1.14	0.1949	0.83	0.54–1.26	0.3857
Etiology	1.25	0.89–1.74	0.1816	1.25	0.88–1.76	0.1971
PS	2.92	2.03–4.20	<0.0001	2.76	1.83–4.17	<0.0001
CPS	1.84	1.15–2.94	0.0101	1.14	0.68–1.92	0.6069
ICI	0.47	0.29–0.79	0.0038	0.64	0.38–1.09	0.1031

Age, age over 75; Sex, male; Etiology, viral hepatitis; PS, performance status more than 1; CPS, Child–Pugh score classified into B; ICI, use of immune check point inhibitor as an initial treatment, *p*-values were calculated using the Cox proportional hazard model; m group, patients with only extrahepatic metastasis.

## Data Availability

Research data related to this study will be made available upon request.
